# Combined quantitative T2 mapping and [^18^F]FDG PET could improve lateralization of mesial temporal lobe epilepsy

**DOI:** 10.1007/s00330-022-08707-5

**Published:** 2022-03-28

**Authors:** Miao Zhang, Hui Huang, Wei Liu, Lihong Tang, Qikang Li, Jia Wang, Xinyun Huang, Xiaozhu Lin, Hongping Meng, Jin Wang, Shikun Zhan, Biao Li, Jie Luo

**Affiliations:** 1 Department of Nuclear Medicine, Ruijin Hospital, Shanghai Jiao Tong University School of Medicine, Shanghai Jiao Tong University, Shanghai, 200240 China; 2 School of Biomedical Engineering, Shanghai Jiao Tong University, Shanghai, 200240 China; 3 Department of Neurosurgery, Ruijin Hospital, Shanghai Jiao Tong University School of Medicine, Shanghai Jiao Tong University, Shanghai, 200240 China; 4 Collaborative Innovation Center for Molecular Imaging of Precision Medicine, Ruijin Center, Shanghai, 200025 China

**Keywords:** Temporal lobe epilepsy, PET/MR, T2 mapping, Lateralization, Hippocampal sclerosis

## Abstract

**Objectives:**

To investigate whether quantitative T2 mapping is complementary to [^18^F]FDG PET in epileptogenic zone detection, thus improving the lateralization accuracy for drug-resistant mesial temporal lobe epilepsy (MTLE) using hybrid PET/MR.

**Methods:**

We acquired routine structural MRI, T2-weighted FLAIR, whole brain T2 mapping, and [^18^F]FDG PET in 46 MTLE patients and healthy controls on a hybrid PET/MR scanner, followed with computing voxel-based *z*-score maps of patients in reference to healthy controls. Asymmetry indexes of the hippocampus were calculated for each imaging modality, which then enter logistic regression models as univariate or multivariate for lateralization. Stereoelectroencephalography (SEEG) recordings and clinical decisions were collected as gold standard.

**Results:**

Routine structural MRI and T2w-FLAIR lateralized 47.8% (22/46) of MTLE patients, and FDG PET lateralized 84.8% (39/46). T2 mapping combined with [^18^F]FDG PET improved the lateralization accuracy by correctly lateralizing 95.6% (44/46) of MTLE patients. The asymmetry indexes of hippocampal T2 relaxometry and PET exhibit complementary tendency in detecting individual laterality, especially for MR-negative patients. In the quantitative analysis of *z*-score maps, the ipsilateral hippocampus had significantly lower SUVR (LTLE, *p* < 0.001; RTLE, *p* < 0.001) and higher T2 value (LTLE, *p* < 0.001; RTLE, *p* = 0.001) compared to the contralateral hippocampus. In logistic regression models, PET/T2 combination resulted in the highest AUC of 0.943 in predicting lateralization for MR-negative patients, followed by PET (AUC = 0.857) and T2 (AUC = 0.843).

**Conclusions:**

The combination of quantitative T2 mapping and [^18^F]FDG PET could improve lateralization for temporal lobe epilepsy.

**Key Points:**

• *Quantitative T2 mapping and*^*18*^*F-FDG PET are complementary in the characterization of hippocampal alterations of MR-negative temporal lobe epilepsy patients.*

• *The combination of quantitative T2 and*^*18*^*F-FDG PET obtained from hybrid PET/MR could improve lateralization for temporal lobe epilepsy.*

**Supplementary Information:**

The online version contains supplementary material available at 10.1007/s00330-022-08707-5.

## Introduction

The majority of drug-resistant epilepsy are mesial temporal lobe epilepsy (MTLE) [1]; MTLE comprises about 80% of epilepsy surgical resections that aim to remove localized epileptogenic zone (EZ) [2]. In MTLE, the epileptogenic zone commonly involves the mesial temporal lobe structures, including the hippocampus, amygdala, and parahippocampal gyrus [3, 4]. The most common finding in EZ of MTLE is hippocampal sclerosis (HS) [4, 5], which is histologically characterized by neuronal loss and gliosis [6–8]. Typical radiological features on magnetic resonance imaging (MRI) of HS include hippocampal atrophy, disrupted internal hippocampal structure, decreased T1-weighted signal, and increased T2-weighted signal [5, 9, 10]. Patients with non-identifiable lesions on MRI (MR-negative) tend to have worse surgical outcomes than patients with MR lesions [11]. Furthermore, over 80% of MR-negative MTLE patients who underwent surgery had abnormal histopathology [12], thus calling for more sensitive imaging techniques.

^18^F-Fluorodeoxyglucose positron emission tomography ([^18^F]FDG PET) has become widely used in presurgical workup of epilepsy, and is recommended by the neuroimaging subcommission of the International League Against Epilepsy (ILAE) [13] despite a lack of correlation between hypometabolism and the severity of either MRI or histopathology [14]. Particularly in MR-negative MTLE patients, FDG PET has been shown to have higher sensitivity in identifying temporal and hippocampal hypometabolism [14, 15], which lead to improved surgical outcome [16]. On the other hand, the detection rate of EZ in epilepsy using FDG PET or FDG PET/CT has been reported to be 36–73% [17, 18], possibly due to subtle or extended hypometabolism.

Quantitative MR T2 relaxometry measures intrinsic tissue property, which may detect subtle pathology in the hippocampus even in the absence of hippocampal atrophy [19–23]. Increased T2 value has been reported to be consistent with histopathologic findings of HS [21, 24–26], which is found to provide more sensitive lesion detection compared to volumetric MRI at 1.5T [21] and T2-weighted fluid-attenuated inversion recovery (FLAIR) at 3.0T [27]. Furthermore, progress in fast imaging techniques allows reliable whole brain quantitative T2 relaxometry using multi-echo spin-echo within clinical feasible time [28]. Thus, T2 mapping might play a role in the lateralization of MTLE in the presurgical evaluation of epilepsy.

Research with hybrid [^18^F]FDG PET/MR system has been conducted in epilepsy patients, which demonstrated higher detection accuracy than PET/CT by fusion of PET images and high-resolution anatomical MR images [18, 29]. Given that T2 relaxometry is sensitive to epileptogenic pathologies, we hypothesize that the combination of [^18^F]FDG PET and T2 mapping would provide the benefit of both metabolic and intrinsic tissue property measurements, which could be manifested both in visual radiological assessments and quantitative analysis for the lateralization of MR-negative MTLE patients.

## Method

### Participants

This study has been approved by the Internal Review Board of Ruijin Hospital. Patients diagnosed with drug-resistant epilepsy between December 2017 and February 2020 were identified. The inclusion criteria of patients are as follows: (1) clinical history, neurological examination, seizure semiologies, scalp video-EEG findings, and neuropsychological deficit pattern that are consistent with the characteristics of unilateral MTLE; (2) MRI was either normal or disclosed patterns suggestive of HS; (3) stereoelectroencephalography (SEEG) examination was obtained to confirm presurgical localization of the EZ. On the other hand, patients with generalized epilepsy syndromes, posttraumatic epilepsy, brain tumors, or other nervous system lesions other than HS were excluded.

We also recruited two healthy control groups. The first control group (group I) consisted of 24 subjects with MRI scans, while the second control group (group II) consisted of 15 subjects with [^18^F]FDG PET/MR. None of the healthy participants had any history of neurologic or psychiatric illness or has taken chronic medications. Written informed consents were obtained from all participants.

### Data acquisition

PET and MRI scans were performed with integrated a 3.0-T hybrid PET/MR scanner (Biograph mMR; Siemens Healthcare). MRI sequences include 3D T1-weighted anatomical images using MPRAGE (resolution 0.5 × 0.5 × 1.0 mm^3^, TR/TE/TI 1900/2.44/900 ms, FOV 250 × 250 mm^2^, 192 slices), T2-weighted FLAIR (resolution 0.4 × 0.4 × 3.0 mm^3^, TR/TE/TI 8460/92/2433 ms, FOV 220 × 220 mm^2^, 45 slices), and a multi-echo spin-echo T2 mapping sequence (in-plane resolution 0.4 × 0.4 mm^2^, 5.0 mm slice thickness, TR/TE_1_/TE_2_/TE_3_/TE_4_/TE_5_/TE_6_ 2000/10.5/21.0/31.5/42.0/52.5/63.0 ms, 21 slices). All patients and controls were administered [^18^F]FDG intravenously using a mean dose of 184.8 ± 29.0 MBq (range 133.2–247.9 MBq), with the scan being initiated 30~50 min after the injection. Static PET data were acquired in a sinogram mode for 15 min, matrix size 344 × 344, and post-filtered with an isotropic full-width half-maximum (FWHM) Gaussian kernel of 2 mm. Attenuation correction was performed using advanced PET attenuation correction with a unique 5-compartment model including bones [30].

### Image processing and analysis

Voxel-wise T2 maps were reconstructed with monoexponential nonnegative least-squares fitting of the multi-echo signals (MapIt; Siemens Medical Solutions). T2 maps were registered to T1-weighted images with the parameters of the registration between the first echo T2-weighted image and T1-weighted image. Meanwhile, voxels with T2 values larger than 170 ms were excluded to alleviate cerebrospinal fluid (CSF) contaminations [31].

The FDG standard uptake value ratios (SUVRs) from PET images were obtained by intensity normalization via global mean scaling to correct individual variations [32]. The SUVR maps were also registered to T1-weighted images. All image registrations were employed with rigid registration (6 degrees-of-freedom) using SPM12 (https://www.fil.ion.ucl.ac.uk/spm/).

#### Voxel-based *z*-score map

The T2 maps and SUVR images of participants were spatially normalized into the Montreal Neurological Institute (MNI) space with the T1-weighted image as the intermediate registration reference. To alleviate individual variations and improve signal-to-noise ratio, all normalized images were smoothed with Gaussian kernel (4 mm FWHM for T2, 8 mm FWHM for SUVR). The *z*-score maps of T2 and SUVR images for patients were computed based on the voxel-wise mean and standard deviation derived from healthy controls. Afterwards, we extracted the *z*-score maps of cerebral cortices and hippocampi using AAL atlas [33], and then map back to the original space with the inverse of the normalization parameters. The processing time is several minutes per subject (pipeline diagram of T2 *z*-score map in Fig. S1).

#### Quantitative hippocampal analysis

The ROIs for bilateral hippocampi were automatically segmented from the T1-weighted image with the FreeSurfer v7.0 package (https://surfer.nmr.mgh.harvard.edu). To reduce partial volume effect, the hippocampal masks were eroded with the standard morphological operation. The resulting hippocampus masks were used to extract hippocampal T2 values and SUVR values.

#### Analysis of the asymmetry

To assess raw asymmetry observed by each hippocampal measurement, we defined the ΔT2_I-C_ and ΔSUVR_I-C_ as ipsilateral (I) minus contralateral (C) hippocampal T2 and SUVR for patients; and ΔT2_L-R_ and ΔSUVR_L-R_ as left (L) to right (R) hippocampal asymmetries.

We further quantified the hippocampal asymmetry, denoted as asymmetry index (AI), based on the corresponding hippocampal *z*-scores. AI_SUVR_ and AI_T2_ were defined as left minus right measurements.

### Physician visual assessment

The MR and PET images were visually analyzed by three experienced radiologists with certificates of both nuclear medicine and radiology. The readers were blinded for the clinical diagnosis of lateralization. The image evaluation was divided in three separate sessions with a break of at least 1 month: (1) routine MR imaging (T1-weighted, FLAIR, and T2-weighted images); (2) T2 (T2 map and *z*-score map); (3) PET (SUVR map and *z*-score map). In the image evaluation process, all cases were presented in a randomized order in every session. Any disagreement was further resolved through discussion.

#### Routine MRI visual assessment

MRI criteria indicative of HS include (1) atrophy of the hippocampus and/or morphology abnormalities of mesial temporal structures on T1-weighted, FLAIR, and T2-weighted images and (2) hyperintensity of the hippocampus or amygdala on FLAIR or T2-weighted images. A patient was classified to be MR-negative if the MRI images look normal; and classified as MR-HS if the MRI images were presented with evidence of HS.

#### T2 mapping visual assessment

Increased signal of the mesial temporal, hippocampus, or amygdala on T2 and T2 *z*-score maps was classified to be positive. The summary of results was used to determine patient’s laterality of T2 mapping.

#### PET visual assessment

PET images were divided into several zones (left and right frontal, temporal, parietal, and occipital lobe) with rainbow grading, and each zone with at least one well-defined PET hypometabolic focus was classified as positive. In PET *z*-score analysis, *z*-score decreasing clusters (hypometabolic zone) were regarded as positive. We combined PET and PET *z*-score results to determine patient’s laterality.

### Statistical analysis

Wilcoxon signed-rank tests were applied to compare hippocampal T2 and SUVR between the left and right hippocampus in healthy control, and between the ipsilateral and contralateral hippocampus in MTLE groups. For group comparisons, Mann–Whitney *U* tests were applied. A *p* value below 0.05 was considered statistically significant.

In order to evaluate the performance of hippocampal asymmetry of PET and T2 in the lateralization of MR-negative MTLE, logistic regression was used to discriminate left and right MR-negative MTLE groups. Left MTLE (LTLE) and right MTLE (RTLE) were defined as positive and negative samples respectively. Due to the limited sample sizes, a leave-one-out cross-validation strategy was implemented to corroborate the predictive generalizability of models. The area under the curve (AUC) of receiver operating characteristic curve (ROC) was calculated to evaluate the performance of each regression model. In addition, mean square error (MSE), the error between the true label and the predicted probability, was calculated to summarize the prediction error of each group. A lower MSE indicates that the classification model is more accurate.

All statistical analyses were performed using IBM SPSS v24. The logistic regression models were performed using the Python scikit-learn (sklearn).

## Results

### Patient demographics

A total of 46 patients (27/19 M/F, age range 14–53 years) were enrolled based on the inclusion and exclusion criteria. The patient demographics are summarized in Table S1. For comparison, healthy control group I (14/10 M/F, age range 14–53 years) for MR scans is age-matched with patients (*p* = 0.76). However, a significant age difference in healthy control group II (7/8 M/F, age range 36–63 years) for FDG PET and patient group was noted (*p* < 0.001).

### Combining quantitative T2 map and [^18^F]FDG PET improves visual assessment

**Fig. 1:**
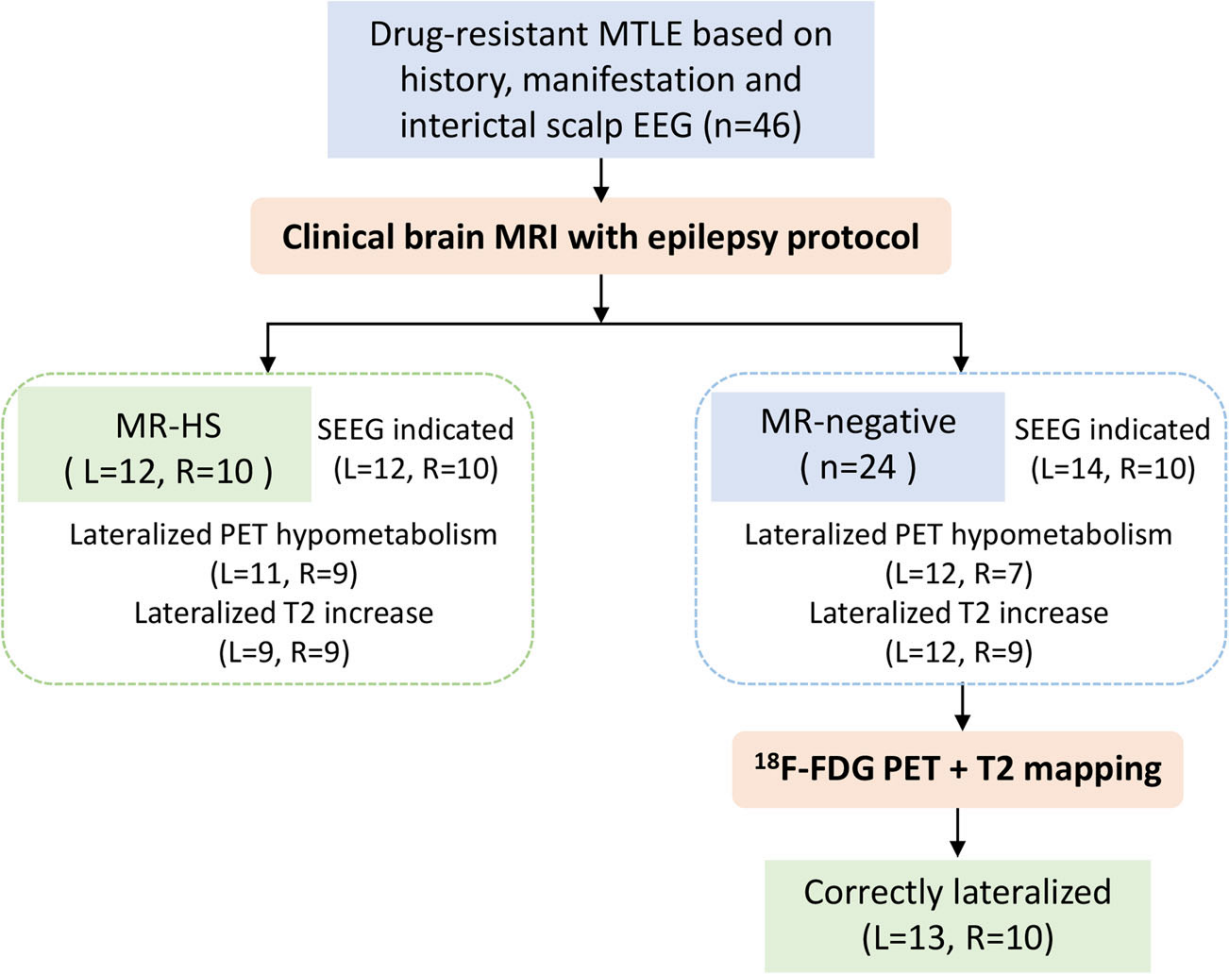
The flowchart of radiological assessment. MTLE, mesial temporal lobe epilepsy; HS, hippocampal sclerosis; L, left; R, right

Among the total 46 patients, there were 24 without visually identifiable hippocampal atrophy or FLAIR hyperintensity, thus MR-negative (Fig. 1). We then divided patients into four groups (MR-HS LTLE; MR-HS RTLE; MR-negative LTLE; MR-negative RTLE) according to standard clinical diagnosis and whether lesions were MR identifiable (Table 1).

**Table 1:** Patient demographics

	MR-HS LTLE	MR-negative LTLE	MR-HS RTLE	MR-negative RTLE
Number of subjects	12	14	10	10
Gender (female/male)	6/6	6/8	4/6	3/7
Age at evaluation (years), median (range)	27 (14–43)	29 (16–44)	26.5 (19–53)	27 (16–46)
Epilepsy duration (years), median (range)	10 (1–17)	4 (1–26)	5.5 (1–30)	10 (1–30)
Seizure frequency (per year), median (range)	27 (6-96)	7 (4–120)	32 (4–120)	18 (4–100)
Postsurgical outcome Engel Class (I/II–IV)/Miss	(8/1)/3	(8/2)/4	(7/3)/0	(6/4)/0

Based on PET hypometabolism, 84.8% were correctly lateralized (20 MR-HS and 19 MR-negative patients) (Table S1). Notably, 4 out of 5 MR-negative patients who had normal glucose metabolism or bilateral hypometabolism in hippocampi and temporal lobes were clearly lateralized in quantitative T2 maps. Individual record of radiological assessment is summarized in Table S1. Hybrid PET/T2 maps correctly lateralized 95.65% (44/46) of patients.

**Fig. 2:**
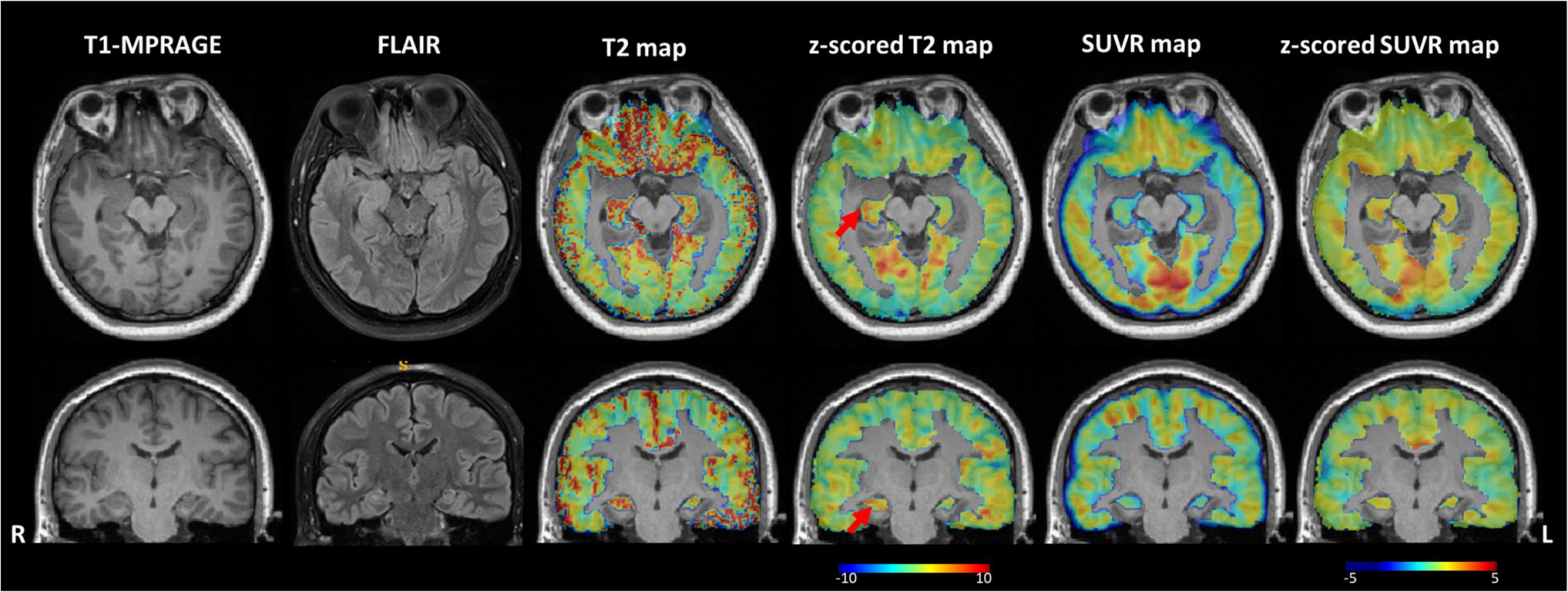
T1-weighted (MPRAGE), FLAIR, PET SUVR, and T2 maps and the *z*-scored maps of a typical MRI-negative RTLE patient, #9. The T1-weighted image, FLAIR image, SUVR, and *z*-score SUVR map have normal findings. T2 map and *z*-scored T2 map show a well-defined increased T2 signal in the right hippocampus and parahippocampus compared with the left hippocampus. SEEG findings indicated the seizure onset originated from the right hippocampus. After right temporal lobe resection, the patient had a follow-up time longer than 1 year, showing Engel class I outcome

In a representative MR-negative RTLE subject (Fig. 2), neither T2w-FLAIR nor FDG PET could lateralize hippocampal damage by visual inspection. The T2 value increase was readily observable in the right hippocampus in quantitative T2 mappings, which was also confirmed by SEEG. This result was also supported by the astrogliosis found in the histopathological stain of the resected mesial temporal cortex of the same patient (Fig. S2).

### Asymmetry indexes of quantitative T2 and FDG uptake are complementary in MTLE lateralization

**Fig. 3:**
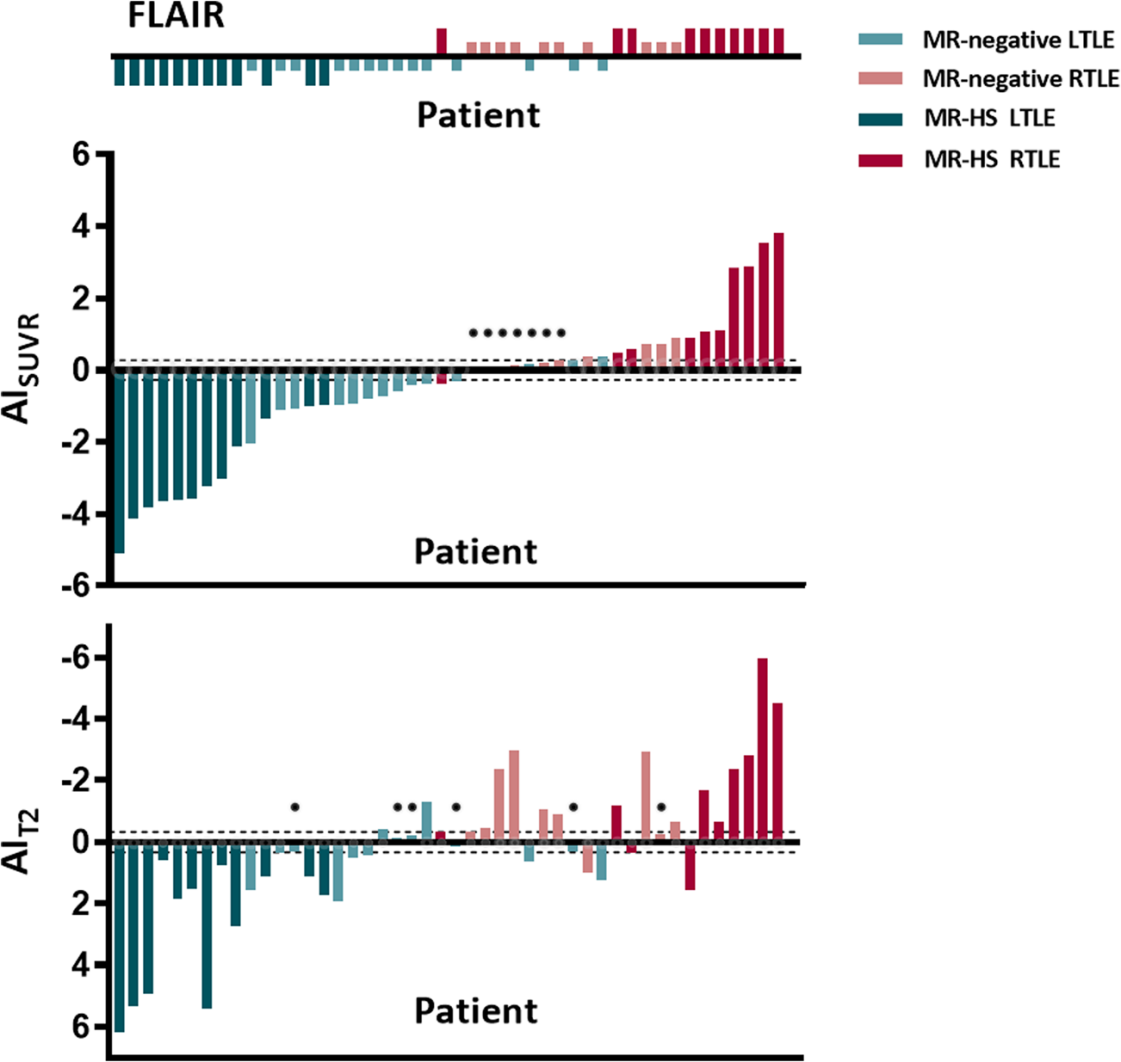
Bar plots of asymmetry index values for each patient. Values shown are left to right hippocampal asymmetry of the *z*-score of SUVR and T2. The patients are arranged in order of AI_SUVR_ from smallest to largest, and the group type of patients by FLAIR diagnosis is shown in the top row. Green is used for left MTLE; Red is used for right MTLE. MR-negative cases are represented by lighter color. The horizontal lines represent range of variations in healthy controls (95% confidence interval)

Figure 3 illustrates hippocampal AIs of both SUVR of FDG uptake and T2 for each patient. The subjects were displayed with ascending AI value of SUVR, negative value being left lateralized and positive value being right lateralized. Heights of AI bars tend to be much shorter for MR-negative patients both in SUVR and T2 value. Among 46 patients, seven MR-negative patients had AI_SUVR_ within the variations of healthy control group (95% confidence interval), and six MR-negative patients had AI_T2_ value within variations of healthy control (95% confidence interval). Notably, no patient had both AI_T2_ and AI_SUVR_ within normal range. Some patients who had very low AI_SUVR_ exhibit much higher AI in T2, and vise versa. This indicated that T2 and PET might potentially have complementary capability in lateralization. Consistent with the visual examination results, four of the five MR-negative patients not properly lateralized based on PET SUVR exhibited obvious T2 asymmetry.

### Statistical analysis of hippocampal alterations and logistic regression models based on PET/T2

**Fig. 4:**
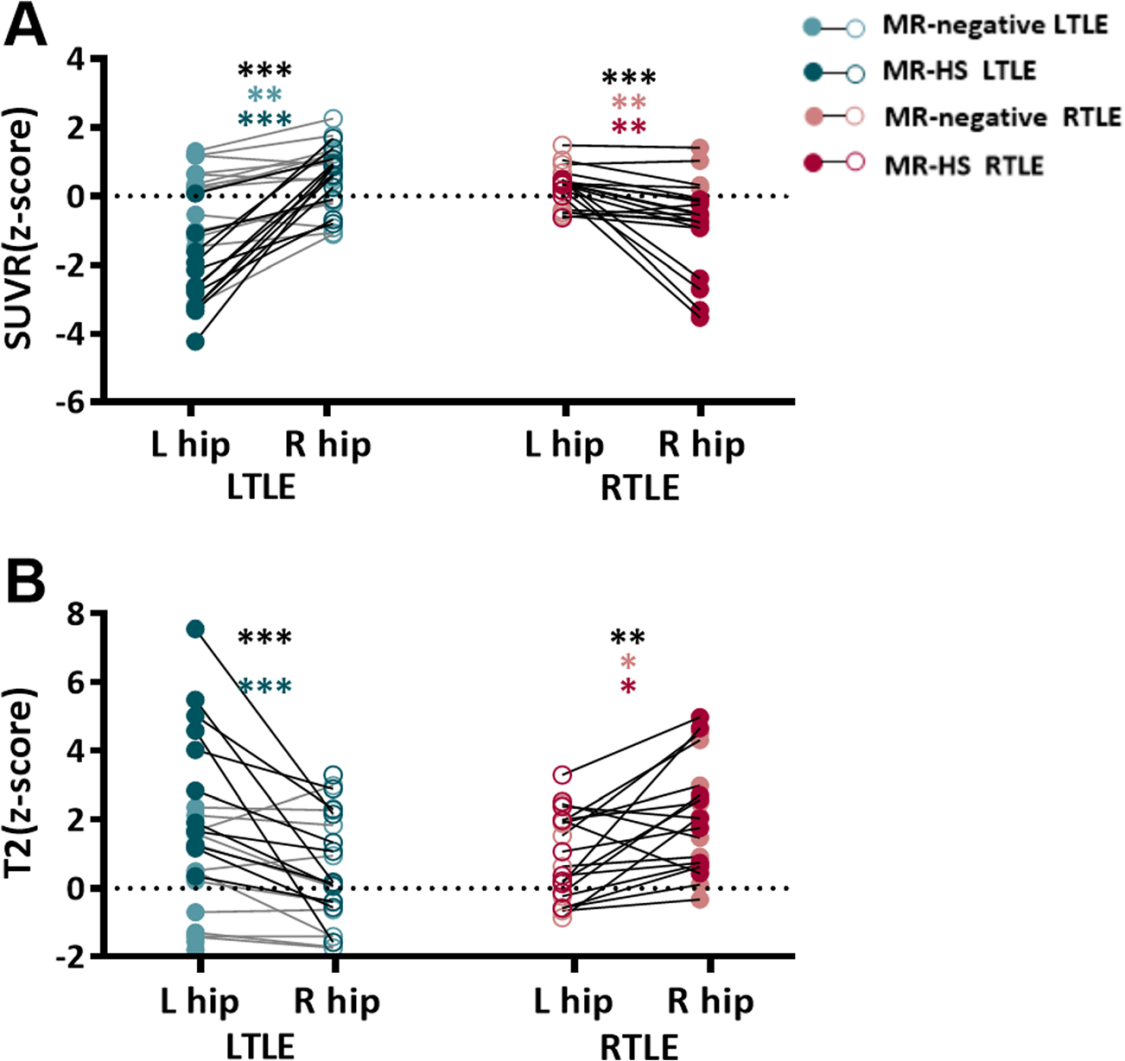
*z*-scored metrics of mean PET SUVR (**A**) and T2 value (**B**) of the left and right hippocampi in different subgroups. Each patient’s ipsilateral (solid dot) and contralateral (hollow dot) hippocampi measurements are connected by a solid line. Green is used for left MTLE; Red is used for right MTLE. MR-negative cases are represented by lighter color. Wilcoxon signed-rank tests are used. *p < 0.05, **p < 0.01, ***p < 0.001

Among the pairwise comparisons between *z*-scored metrics of left and right hippocampi within all LTLE and RTLE, the ipsilateral hippocampus (left hippocampus of LTLE, right hippocampus of RTLE) had significantly lower SUVR (LTLE, *p* < 0.001; RTLE, *p* < 0.001) and higher T2 value (LTLE, *p* < 0.001; RTLE, *p* = 0.001) compared to the contralateral hippocampus (Fig. 4). For MR-negative patients, lower SUVR of the ipsilateral hippocampus in MR-negative LTLE was significant in *z*-score (*p* = 0.002), not raw values (*p* = 0.09). The T2 *z*-score of the ipsilateral hippocampus was significantly higher in MR-negative RTLE (*p* = 0.02), and marginally higher in MR-negative LTLE patients (*p* = 0.06). Taking *z*-score made a difference in statistical significance for T2 compared to raw values (Table 2; Fig. S3).

**Table 2:** The mean difference (left–right) of left and right hippocampus

Subjects group	Raw data			*z*-score		
	Left hip	Right hip	△_L-R_	Left hip	Right hip	△_L-R_
T2						
Healthy control	110.40 ± 3.11	109.23 ± 2.98	1.17 ± 2.38*	0 ± 1.00	0 ± 1.00	0 ± 0.78
MR-negative LTLE	110.86 ± 4.66	108.51 ± 5.01	2.34 ± 2.42**	0.14 ± 1.47	−0.23 ± 1.68	0.38 ± 0.81
MR-HS LTLE	121.81 ± 8.00	111.93 ± 4.50	9.87 ± 6.46***	3.26 ± 2.57	0.88 ± 1.51	2.45 ± 2.08***
MR-negative RTLE	112.93 ± 4.03	114.94 ± 4.96	−2.02 ±3.83	0.81 ± 1.29	1.92 ± 1.67	−1.11 ± 1.28*
MR-HS RTLE	113.89 ± 4.12	117.85 ± 7.48	−3.96 ± 6.72	1.12 ± 1.33	2.90 ± 2.51	−1.78 ± 2.25*
SUVR						
Healthy control	0.93 ± 0.06	0.91 ± 0.07	0.02 ± 0.03**	0 ± 1.00	0 ± 1.00	0 ± 0.54
MR-negative LTLE	0.92 ± 0.07	0.94 ± 0.07	−0.02 ± 0.04	−0.10 ± 1.26	0.51 ± 1.06	−0.61 ± 0.65**
MR-HS LTLE	0.79 ± 0.07	0.95 ± 0.05	−0.16 ± 0.08***	−2.40 ± 1.15	0.56 ± 0.77	−2.97 ± 1.32***
MR-negative RTLE	0.95 ± 0.04	0.91 ± 0.05	0.04 ± 0.02**	0.29 ± 0.74	−0.05 ± 0.79	0.34 ± 0.33**
MR-HS RTLE	0.94 ± 0.02	0.81 ± 0.09	0.13 ± 0.10**	0.22 ± 0.34	−1.47 ± 1.37	1.69 ± 1.45**

Abbreviation: Hip, hippocampus; * *p* < 0.05; **p < 0.01; ***p < 0.001. The *p* values were for paired comparisons between left and right hippocampus. In parentheses are the *p* values that are not statistically significant but may indicate a trend toward being so

In addition, the magnitudes of left/right asymmetry in healthy control subjects (ΔT2_L-R_ = 1.17 ± 2.38 ms, ΔSUVR_L-R_ = 0.02 ± 0.03) were not significantly different from the subtle changes in MR-negative patients (ΔT2_I-C_ = 2.21 ± 3.01 ms, ΔSUVR_I-C_ = −0.03 ± 0.03) in terms of hippocampal T2 (*p* = 0.19) or SUVR (*p* = 0.23), indicating limited ability of lateralization for MR-negative subjects.

**Fig. 5:**
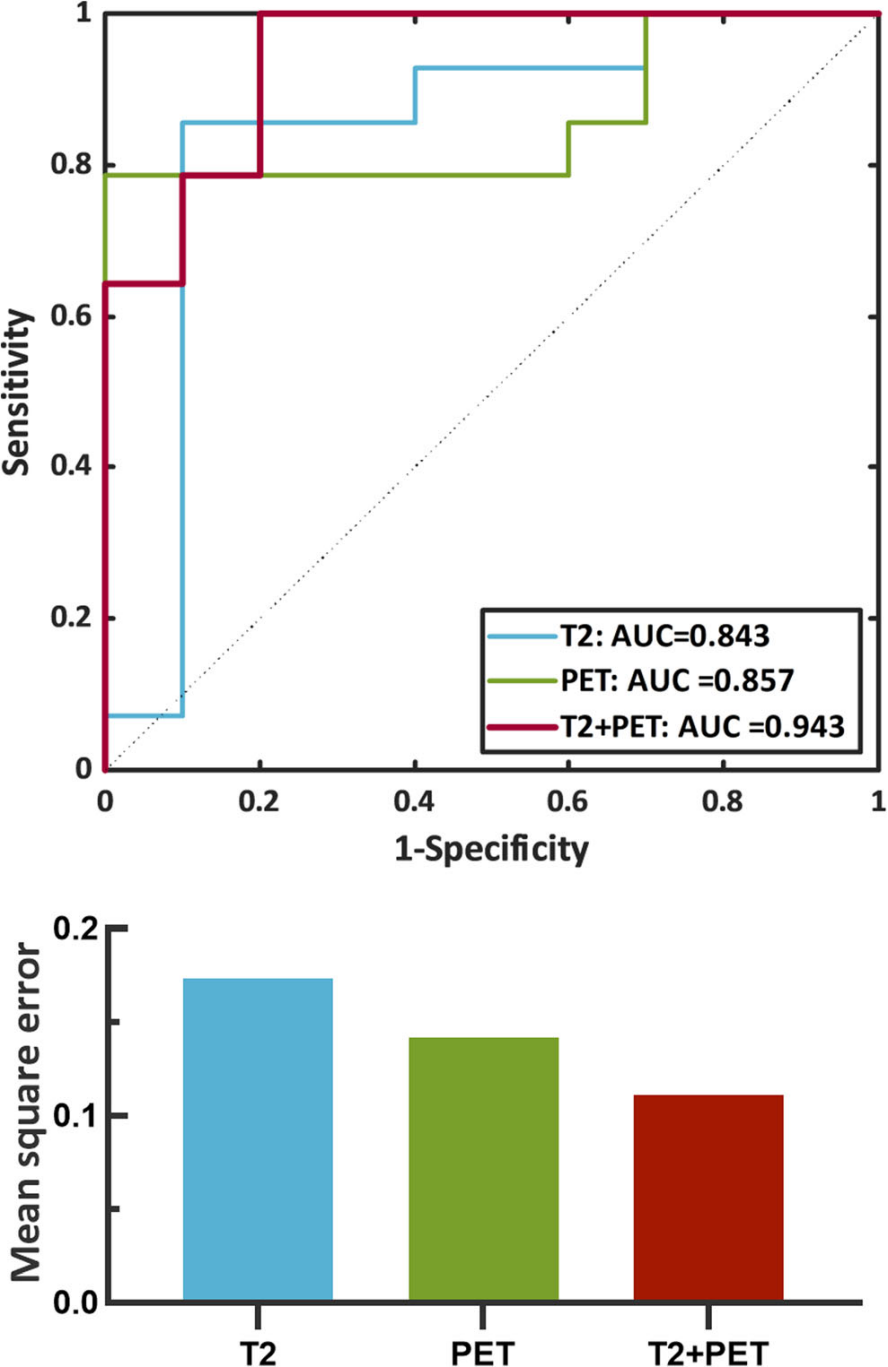
**A** Receiver operating characteristic (ROC) curves of the classification models. **B** Bar plots detailing mean square error from different classification models for MR-negative MTLE subjects

The performance of T2, PET, and combination of T2 and PET (T2+PET) in lateralizing MR-negative MTLE patients was evaluated by logistic regression models, characterized by the ROC curves shown in Fig. 5. The best performance was the multivariable T2+PET models with the highest AUC of 0.943, and lowest MSE of 0.111 (Fig. 5). PET SUVR performs better with the higher AUC of 0.857 and lower MSE of 0.141, followed by T2 (AUC = 0.843, MSE = 0.174).

## Discussions

In this study, we utilized the hybrid PET/MR system to investigate the complementary lateralization capability of FDG PET and T2 mapping for MR-negative MTLE patients. Routine structural MRI and T2w-FLAIR lateralized 47.8% (22/46) of MTLE patients, and T2 mapping combined with [^18^F]FDG PET correctly lateralized 95.6% (44/46) of MTLE patients. For MR-negative patients, FDG PET lateralized 19 out of 24 (79%) by visual assessment (Fig. 1; Table S1). Together with quantitative T2 mapping, 23 out of 24 (95.83%) MR-negative patients were correctly lateralized, suggesting the potential benefit of combining T2 mapping with FDG PET. Our findings indicated that using hybrid classification models based on the combination of these two modalities results in higher AUC in predicting lateralization for MR-negative MTLE patients.

Epileptogenesis is typically characterized by neuronal damage and gliosis [34]. Neuronal damage can lead to hypometabolism and hence may be identified by FDG PET, which was shown to be a promising biomarker for neural injury and dysfunction [35]. However, reactive gliosis in epileptogenesis can also cause partial recovery of glucose hypometabolism [36, 37]. The incidence of hypometabolism recovery from gliosis may further limit the capability of FDG PET in detecting epileptogenesis, which may explain why it did not correlate with histological analysis of tissue damage [14]. On the other hand, strong correlation has been found between T2 signal and histological evidence of gliosis [5, 38]. This may explain why T2 mapping could play a complementary role to FDG PET for the lateralization of EZs in cases when hypometabolism fails to be a good indicator.

Quantitative T2 relaxometry is sensitive to recognize subtle changes, even for epileptogenic hippocampus with normal MR findings [19, 21]. In this work, we also demonstrate that quantitative hippocampal T2 relaxometry can improve lateralization in MTLE patients, which becomes especially valuable for those ambiguous cases in FDG PET. Simultaneous T2 mapping and PET can allow acquisitions under the same physiological and pathophysiological conditions, thus more accurate EZ localization [17]. Compared with single mode modality, T2 mapping combined with [^18^F]FDG PET could improve the lateralization accuracy by correctly lateralizing 95.6% (44/46) of MTLE patients.

Several notes should be taken when considering T2 mapping incorporation into clinical routine. (1) Scan parameters: Considering the multiexponential nature of brain T2 relaxometry [39], difference in acquisition parameter selection could partially contribute to difference in absolute T2 values among literature reports [40]. (2) CSF removal: Because CSF has very long T2, it appears very bright on T2 maps, interfering visual inspections. In this study, we erode the boundary of hippocampal segmentation and employ a threshold of 170 ms to minimize CSF contamination [31]. (3) Quantitative comparisons: We observed longer T2 relaxation time (ΔT2_L-R_ = 1.17 ± 2.38 ms, *p* = 0.04) and slightly higher SUVR (ΔSUVR_L-R_ = 0.02 ± 0.03, *p* = 0.01) in the left hippocampus compared to the right hippocampus in healthy subjects (Supplementary Material 2). The left-right asymmetry of healthy subjects may confound the subtle ipsilateral-contralateral asymmetry of MRI-negative patients, complicating visual assessment of MR-negative cases. As commonly done for FDG PET images, automated quantitative approach of hippocampal asymmetry by *z*-score normalization would avoid the visual bias of having asymmetric baseline T2. Quantitative hippocampal T2 can objectively characterize the presence and laterality of hippocampal abnormalities in MTLE with the accuracy comparable or even better than the performance of visual diagnosis from experts [19, 31, 41]. Finally, with current fast imaging strategy and computation power, whole brain T2 mapping is feasible in clinical scans, with the possibility of incorporating image processing steps into radiological workstation to aid clinical assessments.

This study has limitations. The ages of healthy control for PET are not well-matched with patients. Since glucose metabolism of the hippocampus has been reported as age-independent from age 16 to 80 years [42–44], we did not recruit extra healthy volunteers to radioactive PET imaging. Another limitation is the small sample size, which is why leave-one-out cross-validation was used in testing the classification models, and also due to subpial aspiration during surgery, which is supposed to minimize the damage to surrounding structures during the operation. As a result, it was very challenging to obtain the en bloc resected hippocampal specimens for histopathological studies. Future studies will be designed to collect relatively intact hippocampus specimens and expand larger cohorts to confirm our findings.

## Conclusions

Our study demonstrated the effectiveness of hybrid PET/MR imaging in lateralization of MR-negative MTLE. The synergy of FDG PET and quantitative T2 mapping images was shown to be better characterizing hippocampal abnormalities.

## Supplementary information

ESM 1 (DOCX 1073 kb)

## Abbreviations

[^18^F]FDG PET: ^18^F-fluorodeoxyglucose positron emission tomography
AI: Asymmetry index
AUC: Area under the curve
CSF: Cerebrospinal fluid
EZ: Epileptogenic zone
FLAIR: Fluid-attenuated inversion recovery
FOV: Field of view
Hip: Hippocampus
HS: Hippocampal sclerosis
LTLE: Left temporal lobe epilepsy
MRI: Magnetic resonance imaging
MSE: Mean square error
MTLE: Mesial temporal lobe epilepsy
ROC: Receiver operating characteristic curve
RTLE: Right temporal lobe epilepsy
SEEG: Stereoelectroencephalography
SUVR: Standard uptake value ratio
TE: Echo time
TI: Inversion time
TR: Repetition time

## Declarations

### Guarantor

The scientific guarantor of this publication is Miao Zhang.

### Statistics and biometry

No complex statistical methods were necessary for this paper.

### Methodology

• prospective

• cross sectional study

• performed at one institution
