# Supplementary material for: Combined quantitative T2 mapping and [18F]FDG PET could improve lateralization of mesial temporal lobe epilepsy
Source: Eur Radiol. 2022 Mar 28;32(9):6108–17. doi: 10.1007/s00330-022-08707-5 (PMC9381472; doi:10.1007/s00330-022-08707-5)
Supplement: Supplementary file 1 — (DOCX 1073 kb) [file 330_2022_8707_MOESM1_ESM.docx]

**Table S1.** Individual records of patient demographics, clinical features, seizure localization, postoperative outcomes, and radiological assessment results.

| **ID** | | **Sex** | **Age** | **Age at epilepsy onset,** | **Epilepsy Duration** | **Seizure frequency (per year)** | **SEEG record EZ location** | **Surgery resection extent** | **Outcome**  **Engel class** | **MR Lateralization** | **PET Lateralization** | **T2 mapping Lateralization** | **T2+PET**  **Lateralization** |
| --- | --- | --- | --- | --- | --- | --- | --- | --- | --- | --- | --- | --- | --- |
| 1 | M | | 25 | 9 | 16 | 24 | Right temporal lobe and hippocampus | Right anterior temporal and hippocampus amygdala lobectomy | 1 | N | R | R | R |
| 2 | M | | 28 | 27 | 1 | 6 | Left anterior temporal lobe and hippocampus | Left anterior temporal and hippocampus amygdala lobectomy | missing | N | N | L | L |
| 3 | F | | 20 | 15 | 5 | 120 | Right temporal lobe and hippocampus | Right anterior temporal and hippocampus amygdala lobectomy | 1 | R | R | R | R |
| 4 | M | | 19 | 5 | 14 | 24 | Right temporal lobe and hippocampus | Right anterior temporal and hippocampus amygdala lobectomy | 1 | R | R | R | R |
| 5 | M | | 43 | 40 | 3 | 12 | Left hippocampus and amygdala | Left anterior temporal and hippocampus amygdala lobectomy | missing | L | L | L | L |
| 6 | F | | 17 | 15 | 2 | 6 | Left anterior temporal lobe and hippocampus | Stereotactic deep brain nuclei destruction | 3 | N | L | L | L |
| 7 | M | | 23 | 11 | 12 | 6 | Left anterior temporal lobe and amyloid | Left anterior temporal and hippocampus amygdala lobectomy | 1 | N | N | N | N |
| 8 | M | | 32 | 23 | 9 | 48 | Left anterior temporal lobe and amyloid | Left anterior temporal and hippocampus amygdala lobectomy | 1 | L | L | L | L |
| 9 | M | | 25 | 23 | 2 | 100 | Right temporal lobe and hippocampus | Right anterior temporal and hippocampus amygdala lobectomy | 1 | N | N | R | R |
| 10 | F | | 43 | 39 | 4 | 9 | Left anterior temporal lobe and hippocampus | Left anterior temporal and hippocampus amygdala lobectomy | missing | N | L | L | L |
| 11 | F | | 15 | 12 | 3 | 12 | Left anterior temporal lobe and hippocampus | Left anterior temporal and hippocampus amygdala lobectomy | 1 | N | L | L | L |
| 12 | M | | 19 | 16 | 3 | 6 | Right temporal lobe and hippocampus | Right anterior temporal and hippocampus amygdala lobectomy | 1 | N | R | R | R |
| 13 | M | | 26 | 12 | 14 | 12 | Left anterior temporal lobe and hippocampus | Left anterior temporal and hippocampus amygdala lobectomy | missing | L | L | L | L |
| 14 | F | | 26 | 24 | 2 | 7 | Right hippocampus and amygdala | Right anterior temporal and hippocampus amygdala lobectomy | 1 | R | L | R | R |
| 15 | M | | 14 | 12 | 2 | 12 | Left hippocampus and amygdala | Left anterior temporal and hippocampus amygdala lobectomy | missing | L | L | L | L |
| 16 | M | | 23 | 13 | 10 | 90 | Right temporal lobe and hippocampus | Right anterior temporal and hippocampus amygdala lobectomy | 1 | R | R | R | R |
| 17 | M | | 16 | 13 | 3 | 12 | Left anterior temporal lobe and hippocampus | Left anterior temporal and hippocampus amygdala lobectomy | missing | N | L | L | L |
| 18 | F | | 27 | 13 | 14 | 24 | Left anterior temporal lobe and amyloid | Left anterior temporal and hippocampus amygdala lobectomy | 1 | L | L | L | L |
| 19 | M | | 31 | 20 | 11 | 12 | Right temporal lobe | Right anterior temporal and hippocampus amygdala lobectomy | 4 | R | R | R | R |
| 20 | F | | 29 | 26 | 3 | 4 | Left temporal lobe and hippocampus | Left anterior temporal and hippocampus amygdala lobectomy | 2 | N | L | L | L |
| 21 | M | | 32 | 31 | 1 | 6 | Right temporal lobe and hippocampus | Right anterior temporal and hippocampus amygdala lobectomy | 1 | N | R | R | R |
| 22 | M | | 45 | 38 | 7 | 6 | Right temporal lobe and hippocampus | Right anterior temporal and hippocampus amygdala lobectomy | 1 | R | R | R | R |
| 23 | F | | 23 | 20 | 3 | 30 | Left temporal lobe and hippocampus | Left anterior temporal and hippocampus amygdala lobectomy | 1 | L | L | B | L |
| 24 | F | | 22 | 21 | 1 | 4 | Left anterior temporal lobe and amyloid | Left anterior temporal and hippocampus amygdala lobectomy | missing | N | L | L | L |
| 25 | M | | 30 | 20 | 10 | 48 | Left hippocampus and amygdala | Left anterior temporal and hippocampus amygdala lobectomy | 1 | L | L | B | L |
| 26 | F | | 16 | 14 | 2 | 30 | Right temporal lobe and hippocampus | Right anterior temporal and hippocampus amygdala lobectomy | 2 | N | R | B | R |
| 27 | F | | 42 | 12 | 30 | 5 | Right anterior temporal lobe and hippocampus | Right anterior temporal and hippocampus amygdala lobectomy | 4 | N | R | R | R |
| 28 | M | | 30 | 29 | 1 | 6 | Left anterior temporal lobe and amyloid | Left anterior temporal and hippocampus amygdala lobectomy | 1 | N | L | L | L |
| 29 | M | | 29 | 19 | 10 | 120 | Left temporal lobe and hippocampus | Selective amygdala hippocampus resection and Stereotactic deep brain nuclei destruction | 1 | N | L | L | L |
| 30 | F | | 27 | 17 | 10 | 24 | Left hippocampus and amygdala | Stereotactic target lesion damage in the brain (left amygdala) | 4 | L | L | L | L |
| 31 | M | | 22 | 5 | 17 | 48 | Left hippocampus and amygdala | Left anterior temporal and hippocampus amygdala lobectomy | 1 | L | L | L | L |
| 32 | M | | 29 | 24 | 5 | 70 | Left anterior temporal lobe and hippocampus | Left anterior temporal and hippocampus amygdala lobectomy | 1 | N | L | L | L |
| 33 | F | | 53 | 36 | 17 | 24 | Right hippocampus and amygdala | Right anterior temporal and hippocampus amygdala lobectomy | 1 | R | R | R | R |
| 34 | M | | 19 | 11 | 8 | 96 | Right temporal lobe and hippocampus | Right anterior temporal and hippocampus amygdala lobectomy | 1 | N | R | R | R |
| 35 | M | | 28 | 18 | 10 | 40 | Right temporal lobe | Right anterior temporal and hippocampus amygdala lobectomy | 2 | R | R | R | R |
| 36 | F | | 36 | 22 | 14 | 96 | Left hippocampus and amygdala | Left anterior temporal and hippocampus amygdala lobectomy | 1 | L | B | B | B |
| 37 | F | | 37 | 34 | 3 | 30 | left anterior temporal lobe and hippocampus | Light temporal lesion ablation | 1 | N | L | L | L |
| 38 | M | | 34 | 14 | 20 | 12 | Left anterior temporal lobe and amyloid | Left anterior temporal and hippocampus amygdala lobectomy | 1 | N | L | N | L |
| 39 | M | | 29 | 27 | 2 | 4 | Right temporal lobe | Right anterior temporal and hippocampus amygdala lobectomy | 1(4) | N | N | R | R |
| 40 | M | | 44 | 18 | 26 | 7 | Left anterior temporal lobe and amyloid | Left anterior temporal and hippocampus amygdala lobectomy | 1 | N | L | L | L |
| 41 | M | | 20 | 8 | 12 | 96 | Right temporal lobe and hippocampus | Right anterior temporal and hippocampus amygdala lobectomy | 1 | R | R | R | R |
| 42 | F | | 27 | 21 | 6 | 96 | Right hippocampus and amygdala | Right anterior temporal and hippocampus amygdala lobectomy | 4 | R | R | L | R |
| 43 | F | | 46 | 33 | 13 | 12 | Right anterior temporal lobe and hippocampus | Right anterior temporal and hippocampus amygdala lobectomy | 1 | N | R | R | R |
| 44 | F | | 27 | 17 | 10 | 30 | Left anterior temporal lobe and amyloid | Left anterior temporal and hippocampus amygdala lobectomy | 1 | L | L | L | L |
| 45 | M | | 31 | 16 | 15 | 96 | Right temporal lobe | Right anterior temporal and hippocampus amygdala lobectomy | 3 | N | B | R | R |
| 46 | F | | 39 | 29 | 10 | 6 | Left anterior temporal lobe and hippocampus | Left anterior temporal and hippocampus amygdala lobectomy | 1 | L | L | L | L |

Abbreviation: L, left lateralized abnormalities; R, right lateralized abnormalities; B, Bilateral abnormalities; N, normal findings.

**Supplementary Figure 1**


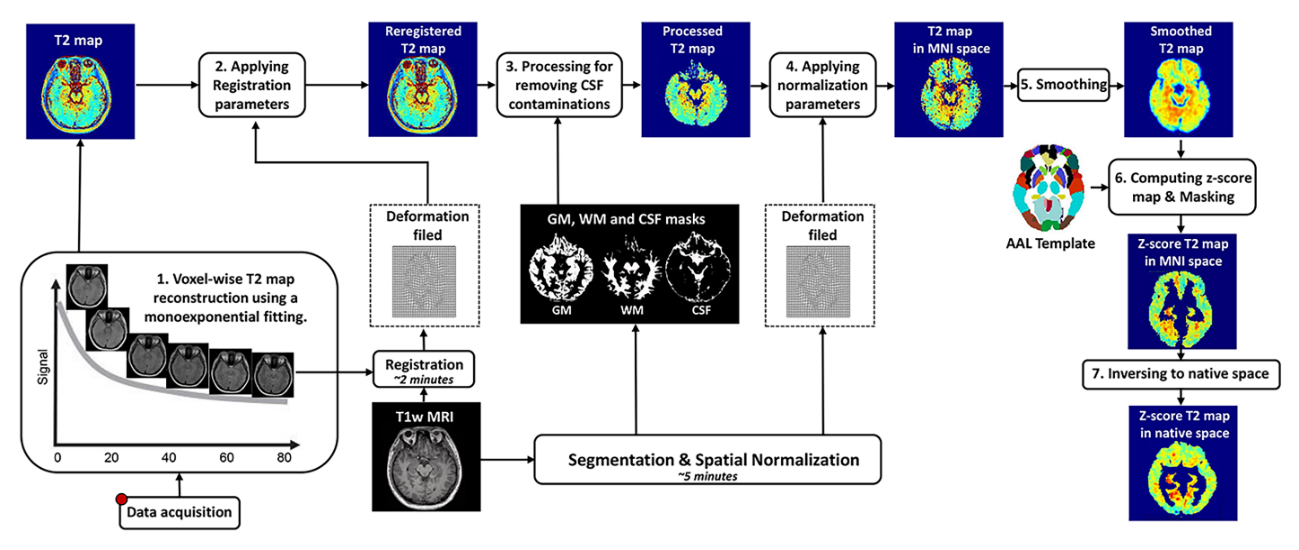


**Figure S1.** Post-processing pipeline of voxel-based T2 z-score maps. Step 1: The voxel-wise T2 maps were reconstructed with monoexponential nonnegative least-squares fitting; Step 2: T2 maps were registered to T1-weighted image with the parameters of the registration between the first echo T2-weighted image and T1-weighted image; Step 3: Registered T2 maps were masked to remove the region covered by CSF, and voxels with T2 values larger than 170 ms were excluded, to alleviate CSF contaminations; Step 4: The processed T2 maps were then warped into MNI space using parameters from unified segmentation of T1-weighted image; Step 5: The normalized images were smoothed with 4 mm FWHM Gaussian kernel; Step 6: The T2 z-score map of individual epilepsy patient was computed with respect to the distribution of healthy control group; Step 7: The T2 z-score maps were put back to the native space with the inverse of the normalization parameters. Note that the most time-consuming parts are rigid body image registrations (~2 minutes) and the unified segmentation and normalization procedure (~5 minutes), which can be done using freely available SPM12.

**Supplementary Figure 2**

**
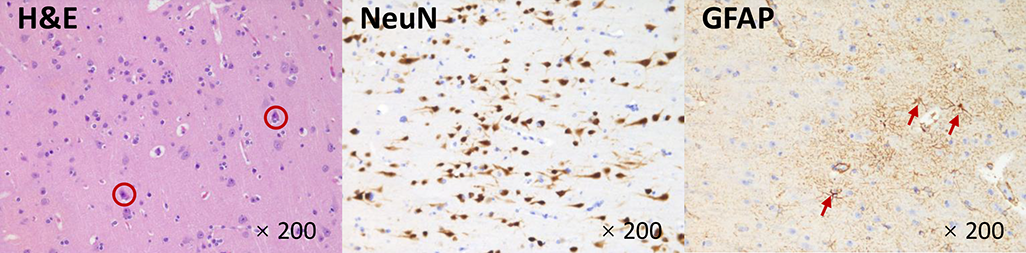
**

**Figure S2.** Representative histological stains of mesial temporal cortex of patient #9 with MR-negative RTLE. The tissue specimens were stained with hematoxylin and eosin (H&E), and immunostained with NeuN (neuron nuclei [NeuN] for neurons) and GFAP (glial fibrillary acidic protein [GFAP] for reactive astrocytes). Dysmorphic neurons are highlighted by red circles shown in H&E stains. Increased astrogliosis are highlighted by red arrows in GFAP stains.

**Supplementary Figure 3**


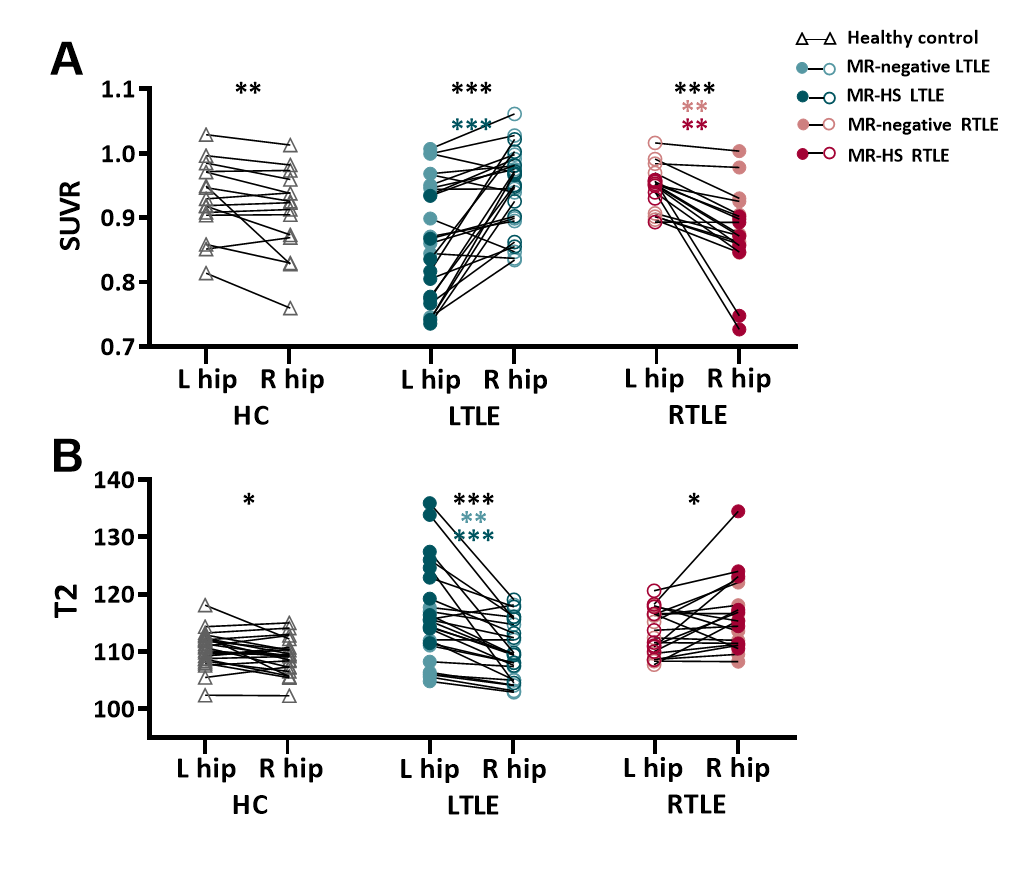


**Figure S3.** Raw values of mean PET SUVR (A) and T2 value (B) of left and right hippocampi in different subgroups. Wilcoxon signed-rank tests were used to compare the difference between the left and right hippocampus of healthy control, and the difference between the ipsilateral hippocampus and the contralateral hippocampus of the MR-HS and MR-negative patients groups. * *P* < 0.05, ** *P* < 0.01, *** *P* < 0.001.
